# Insect frass composition and potential use as an organic fertilizer in circular economies

**DOI:** 10.1093/jee/toad234

**Published:** 2024-01-03

**Authors:** Helen C S Amorim, Amanda J Ashworth, Komala Arsi, M Guadalupe Rojas, Juan A Morales-Ramos, Annie Donoghue, Kelsy Robinson

**Affiliations:** USDA-ARS Poultry Production and Product Safety Research Unit, Fayetteville, AR 72701, USA; Crop, Soil, and Environmental Sciences, University of Arkansas, Fayetteville, AR 72701, USA; USDA-ARS Poultry Production and Product Safety Research Unit, Fayetteville, AR 72701, USA; USDA-ARS Poultry Production and Product Safety Research Unit, Fayetteville, AR 72701, USA; USDA-ARS National Biological Control Laboratory, Biological Control of Pests Research, Stoneville, MS 38776, USA; USDA-ARS National Biological Control Laboratory, Biological Control of Pests Research, Stoneville, MS 38776, USA; USDA-ARS Poultry Production and Product Safety Research Unit, Fayetteville, AR 72701, USA; USDA-ARS Poultry Research Unit, Mississippi State, MS 39759, USA

**Keywords:** value-added product, animal manure, nutrient supply, sustainable intensification, product safety

## Abstract

Insect manure or “frass” has emerged as an alternative nutrient source for alleviating the dependence on fossil fuel-based fertilizers, reducing food waste, and promoting food security. Yet, research on insect frass chemical composition is in its infancy. Here, we assessed the chemical properties of yellow mealworm (*Tenebrio molitor* L.) frass compared with poultry litter (PL). Insect frass was obtained from the National Biological Control Laboratory (NBCL; IF-L) and an insect-rearing company (IF-C). PL was collected from facilities in Arkansas (PL-AR) and North Carolina (PL-NC). Samples were analyzed for pH, electrical conductivity, macro- and micronutrients, heavy metals, pathogens, and indicator microorganisms. On average, insect frass had 43% and 47% higher C and N than PL, respectively (*P* < 0.05). Considering a 5 mg/ha application rate, IF-C can supply 159 kg N/ha, twice the N supply of PL-AR (78 kg/ha). IF-L had a 53% higher P supply than PL-NC. Mean K, Ca, S, and micronutrient contents were higher in PL than in frass (*P* < 0.05), whereas As, Cd, Cr, and Pb were nearly absent in frass. Chemical composition and pathogens in fertilizer sources were largely affected by insect-rearing substrate and supplements used in poultry and insect production. Insect frass utilized in this study had optimum C and N rates relative to PL, suggesting a promising soil amendment for improving soil health and C sequestration, thus contributing to sustainable agricultural intensification and reuse of food waste in circular economies.

## Graphical Abstract

**Graphical Abstract:**
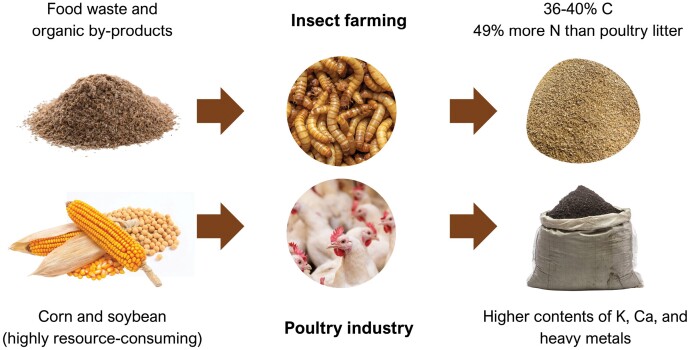

## Introduction

Global food demand is projected to increase 70% by 2050 owing to the economic and population growth of developing countries. As income grows, food consumption shifts toward a greater demand for meat and dairy products, which are highly resource-consuming industries (FAO 2018a, Michalk et al. 2018). Currently, livestock occupies nearly 30% of the global land area (3.5 billion ha) as grazing lands and 33% of global cropland for feed production (Tabassum-Abbasi and Abbasi 2016, FAO 2018b). The increasing demand for animal-based protein may be followed by the expansion of new land and intensification of agricultural practices, which may result in negative impacts on the environment and risk the long-term sustainability of livestock production (Michalk et al. 2018). The search for alternative protein sources for both animal feed and human consumption is, therefore, highly needed.

Insect farming or “mini livestock” is the practice of rearing insects for animal feed production or human consumption and has gained attention in recent years. Compared with conventional livestock, insect farming is a more environmentally friendly industry, owing to much lower demand for land and water, lower greenhouse gas emissions, and enhanced feed conversion efficiency (van Huis and Oonincx 2017). Moreover, a number of insect species can be fed food waste and organic byproducts, yielding a high-value protein that can be used as animal feed (Makkar et al. 2014) or fed directly to humans. This is particularly relevant, considering that 17% of the global agricultural products (931 million tonnes) are wasted every year, 2/3 at the household level (UNEP 2021). Thus, insect farming offers an opportunity to mitigate the environmental impacts of animal feed production, while reducing food waste and enhancing global food security.

A primary byproduct of insect rearing is insect frass, a mixture of excreta and molted skins. This material has increased nutrient contents, which may vary with insect species and substrate composition (Poveda 2021, Beesigamukama et al. 2022), and has an enormous potential to be used as an organic fertilizer. A recent study (Beesigamukama et al. 2022) demonstrated frass of black soldier fly (*Hermetia illucens* L.) had the highest total N (3%) and K (4%) contents and promoted increased seed germination (>90%) compared with other insect species. Frass of mealworm [*Tenebrio molitor* L. (Coleoptera: Tenebrionidae)], containing 5% total N, 1.7% K, and 2% P had mineralization rates and agronomic efficiency comparable to conventional synthetic mineral fertilizer (Houben et al. 2020). Still, N and P speciation and availability, as well as the presence of pathogens and heavy metals, need to be determined to ensure the safe use of insect frass in agriculture.

Using insect frass fertilizer as an alternative source of nutrients would alleviate the dependence on mineral and synthetic fertilizers and potentially reduce costs of agricultural production. Additionally, the reuse of organic byproducts as insect feed contributes to a circular economy (Velenturf and Purnell 2021) and more sustainable production (https://sdgs.un.org/goals/goal12). Yet, the use of insect frass fertilizer in agriculture is novel, and research on frass composition is still incipient, particularly compared with other commonly used animal manures (e.g., poultry litter [PL]). Characterization of physiochemical properties of insect frass is the first step to understand its behavior in soils and potential benefits to soil–plant systems, as well as to provide information to support and regulate its use as an organic fertilizer. Thus, this study aimed to assess the physical and chemical properties and potential nutrient supply of insect frass from varying sources compared with poultry litter (PL). We hypothesized that (i) insect frass has higher nutrient content than PL and that (ii) contents of heavy metals and pathogens are lower in insect frass compared with PL.

## Materials and Methods

### Insect Rearing and Frass Collection

Colony stock of mealworms *Tenebrio molitor* L. (Coleoptera: Tenebrionidae) used in this study was described by Morales-Ramos et al. (2012, 2019). The colony was established from larvae originating from Southeastern Insectaries (Perry, GA) and has been in culture at the USDA ARS National Biological Control Laboratory (NBCL; Stoneville, MS) laboratory since 2015. Larvae were fed with a diet consisting of 90% wheat bran and 10% dry diced potato cubes. Adults received a dry diet consisting of 73% wheat bran, 16.2% potato flour, 3% rice bran, 2.4% dry egg whites, and 0.4% soy flour. Dry ingredients were mixed and ground in a food processor. The resulting powder was transferred to a stainless-steel bowl and 60% reverse osmosis water was added. The paste was homogenized using a regular mixer and spread onto a nonstick pan to dry in a vacuum oven (50 °C and 20 psi for 72 h). Both adults and larvae were kept in a control environment room at 28 ± 5 °C, 60%–75% relative humidity, and 0L:24D (dark) conditions. Water was provided only to the adults twice a week using a spray bottle. Rearing hardware and protocols were followed as described by Morales-Ramos et al. (2012, 2019).

Approximately 7,000 five-wk-old larvae were transferred to a fiberglass stackable tray (41 × 62.5 × 15 cm W × L × H). The bottom of each tray was modified to securely fit a nylon screen standard mesh No. 35 with a 500 µm opening. The trays were stacked in series of 5, with the oldest larvae placed in the bottom tray. Wheat bran and potato mix was added once a week as needed. Natural larvae movement allowed the frass to fall by gravity to the lowest tray. Frass was weekly collected from the tray located at the bottom of the stack (Morales-Ramos et al. 2012). The larvae did not receive water; thus, the collected frass was dry. The frass obtained at NBCL (IF-L) was weighed and placed in plastic bags for storage in a cool room at 4 °C and 40% relative humidity.

Similarly, frass samples were obtained from an insect-rearing company (Beta Hatch, Cashmere, WA), hereafter named IF-C. Larvae of *T. molitor* were fed with wheat bran as a base diet and apple cores once a week. Production conditions and frass collection were similar to those of NBCL. All methods were conducted in accordance with the protocols recommended by the Institutional Animal Care and Use Committee (IACUC).

### PL Production and Collection

PL was obtained from production facilities in Arkansas (PL-AR) and North Carolina (PL-NC), following Katuwal et al. (2023). Briefly, in Arkansas (University of Arkansas Agricultural Research Station, Fayetteville, AR), production conditions consisted of 50 broiler chicks per pen (2.1 × 1.8 m^2^), reared for 42 days. The bedding material consisted of pine wood shaving (17.5 kg per pen, 5 cm depth) on concrete floors and was used for rearing 3 flocks of birds without the addition of new material. During the rearing period, broilers received feed consisting of corn (64.2%), soybean meal (27.7%), meat and bone meal (2.5%), poultry oil (2.65%), sodium chloride (0.31%), sodium bicarbonate (0.05%), limestone (0.74%), dicalcium phosphate (1%), vitamins, amino acids, trace metals, xylanase, and phytase (Anderson et al. 2021). As such, the litter was a mixture of manure, bedding material, spilled feed, and other wastes from the birds. After rearing 3 flocks of birds, litter samples were collected from different areas within a pen and replicated across 4 pens. Then, samples were homogenized and refrigerated until analyzed.

Similarly, PL-NC samples were collected from several locations in a commercial farm (~1 bird per square feet). Birds were fed with commercial feed (about 65%–70% ground corn, 20%–25% soybean meal, with the remaining including fat, salt, vitamins, minerals, dicalcium phosphate, and amino acids). Pine shavings were used as the bedding material. The caked litter was cleaned out after every flock of birds (about 9 wk). Then, litter samples were refrigerated until further analyses.

### Elemental Characterization

PL and insect frass were analyzed in triplicates. Total C and N were determined by combustion using a Vario Max CN analyzer (Elementar Americas Inc., Ronkonkoma, NY, USA). Soil pH and electrical conductivity (EC) were measured on a 1:10 (soil:water) sample extraction (Self-Davis and Moore 2000). Nitrate-N (NO_3_-N), ammonium- (NH_4_-N), and soluble reactive phosphorus (SRP) were determined on 1:10 litter/water extraction following filtration through a 0.45-µm filter paper (Self-Davis and Moore 2000) by colorimetric analysis on a Skalar auto-analyzer (Skalar, Analytical B.V., AA Breda, The Netherlands). Nitrate-N was analyzed by Cd-reduction (APHA 1992). NH_4_-N was analyzed by salicylate-nitroprusside (USEPA 1979) and SRP by the Murphy and Riley (1962) method. Organic N was calculated as the difference between total N and sum of the inorganic N forms (NH_4_-N and NO_3_-N). Extractable metals (Al, As, Ca, Cd, Cr, Fe, K, Mg, Mn, Na, P, Pb, S, and Zn) were determined on oven-dried insect frass and PL samples by inductively coupled optical emission spectroscopy (ICP-OES) on an Agilent 5110 ICP-OES (Agilent Technologies, Santa Clara, CA, USA), after digestion with HNO_3_ and H_2_O_2_ (Zarcinas et al. 1987).

### Pathogen Enumeration of Insect Frass and PL Samples

Insect frass (IF-L and IF-C) and PL samples from Arkansas (PL-AR) were weighed and homogenized with buffered phosphate diluent in a stomacher for 1 min and allowed to rest for 1 min. Then, 10-fold dilutions were made, and duplicate samples were plated onto different petrifilms or specific media for enumeration of total aerobic bacteria (3M Petrifilm Aerobic Count Plate), total Coliforms (3M Petrifilm Rapid Coliform Count Plates), *Escherichia coli* (3M Petrifilm Rapid *E. coli/Coliform* Count Plates), *Listeria* (3M Petrifilm Environmental Listeria Plates), *Salmonella* (XLD agar, HiMedia Laboratories, Thane West, Maharashtra, India), *Campylobacter* (Campylobacter Line Agar, Line 2001), yeast and mold (3M Petrifilm Yeast and Mold Count Plate), and lactic acid bacteria (3M Petrifilm Lactic Acid Bacteria Count Plate).

### Statistical Analyses

Principal component analysis (PCA) and Pearson’s correlation were performed using the *prcomp* function from the *ggfortify* package and the *corr.test* function, respectively (R Software 4.0.3; R Core Team 2022) to examine the relationships among the elemental composition of insect frass and PL. Analysis of variance (ANOVA) of insect frass and PL elemental composition and potential nutrient supply (response variables) was performed using the SAS MIXED procedure (SAS Institute 2017). Fertilizer source (IF-L, IF-C, AR-PL, and NC-PL) was considered the main effect (explanatory variable) and replicate samples (*n* = 3) as random in a completely randomized design. When significant effects were found, mean separations were performed by the SAS macro “pdmix800” (Saxton 1998), with Fisher’s least significant difference and Type I error rate of 5%. Counts of indicator microorganisms and foodborne pathogens were logarithmically transformed and expressed as Log_10_ colony-forming units per gram (Log_10_ CFU/g). Then, bacterial counts (response variable) were analyzed in a completely randomized design using a 1-way ANOVA followed by Tukey’s post hoc test on GraphPad Prism (version 9.1), considering fertilizer source (IF-L, IF-C, AR-PL, and NC-PL) as the main effect (explanatory variable) and replicate samples (*n* = 3) as random.

## Results

### Nutrients and Heavy Metals

**Fig. 1.**
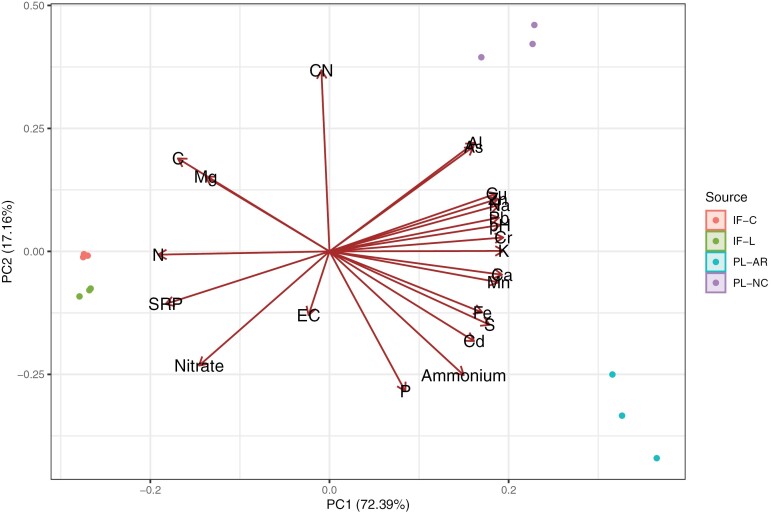
Principal component (PC) biplot of the chemical composition of insect frass samples (*n* = 3) obtained from an insect-rearing company (IF-C), the USDA ARS National Biological Control Laboratory (IF-L), and PL samples collected in Arkansas (PL-AR) and North Carolina (PL-NC). CN, carbon-to-nitrogen ratio.

PCA demonstrated that elemental composition was affected by fertilizer sources and production conditions (Fig. 1), with insect frass samples having similar composition and contrasting with both AR and NC PL samples. Principal components 1 and 2 accounted for 72% and 17% of the total variance, respectively. Carbon, N, NO_3_-N, Mg, and SRP contents were positively correlated between themselves (*r* = 0.63 to 0.91; Supplementary Table 1) and negatively correlated with pH and other macro- and micronutrients and PTEs (*r* = −0.66 to −0.99; Supplementary Table 1). Available P contents were positively correlated with Ca (*r* = 0.52), Fe (*r *= 0.66), and NH_4_-N (*r* = 0.75), but negatively with C (*r* = −0.77).

Chemical composition was affected by fertilizer source and production conditions (Supplementary Table 2). Insect frass had a lower pH than PL (Table 1), which is linked to the increased contents of basic cations (e.g., Ca, K, and Na) (Ashworth et al. 2020). EC was the highest in IF-C, with IF-L and PL-NC having the lowest EC values.

**Table 1.** Mean values (*n* = 3) ± standard error of pH, EC, contents of macro- and micronutrients, and potentially toxic elements (PTEs) in insect frass samples obtained from an insect-rearing company (IF-C), the USDA ARS National Biological Control Laboratory (IF-L) and poultry litter samples collected in Arkansas (PL-AR) and North Carolina (PL-NC)

Chemical properties^a^	Fertilizer source			
	IF-C	IF-L	PL-AR	PL-NC
pH	6.08 ± 0.2b^b^	6.39 ± 0.2b	8.72 ± 0.2a	8.64 ± 0.2a
EC (mS/cm)	9.89 ± 0.6a	4.35 ± 0.4c	7.64 ± 0.6b	5.01 ± 0.6c
C (%)	39.7 ± 0.6a	35.6 ± 0.4b	20.6 ± 0.6d	32.3 ± 0.6c
C:N ratio	11.2 ± 0.5b	10.5 ± 0.3 bc	9.4 ± 0.5c	13.1 ± 0.5a
N (%)	3.5 ± 0.1a	3.4 ± 0.1b	2.2 ± 0.1d	2.5 ± 0.1c
NH_4_-N (mg/kg)	1420.2 ± 43c	1289.9 ± 43c	6123.6 ± 43a	1835.6 ± 43b
NO_3_-N (mg/kg)	41.8 ± 3.4a	46.3 ± 3.4a	27.2 ± 3.4b	3.8 ± 3.4c
Organic N (%)	3.4 ± 0.1a	3.3 ± 0.1a	1.6 ± 0.1c	2.3 ± 0.1b
P (%)	1.6 ± 0.1c	1.9 ± 0.1b	2.2 ± 0.1a	1.6 ± 0.1c
SRP (%)	0.8 ± 0.01b	1.1 ± 0.01a	0.17 ± 0.01c	0.07 ± 0.01d
K (%)	1.9 ± 0.1c	1.9 ± 0.1c	3.7 ± 0.1a	3.3 ± 0.1b
Ca (%)	0.1 ± 0.04c	0.1 ± 0.03c	3.3 ± 0.04a	2.2 ± 0.04b
Mg (%)	0.7 **±** 0.02a	0.7 ± 0.01a	0.6 ± 0.02b	0.7 ± 0.02ab
S (%)	0.3 ± 0.01c	0.3 ± 0.01d	0.9 ± 0.01a	0.6 ± 0.01b
Al (mg/kg)	15.5 ± 17c	4.8 ± 12c	309.2 ± 17b	586.7 ± 17a
Na (mg/kg)	260.5 ± 115c	92.4 ± 81c	6250.0 ± 115b	6866.7 ± 115a
Cu (mg/kg)	16.8 ± 13.3c	17.9 ± 9.4c	530.0 ± 13.3b	636.7 ± 13.3a
Fe (mg/kg)	70.7 ± 21c	87.3 ± 15c	274.0 ± 21a	163.2 ± 21b
Mn (mg/kg)	242.3 ± 8c	165.9 ± 6d	608.3 ± 8a	441.5 ± 8b
Zn (mg/kg)	133.9 ± 11c	107.9 ± 8c	606.7 ± 11b	680.0 ± 11a
As (mg/kg)	0 ± 0c	0 ± 0c	0.7 ± 0.1b	1.3 ± 0.1a
Cd (mg/kg)	0.2 ± 0.1bc	0.2 ± 0.1c	0.7 ± 0.1a	0.3 ± 0.1b
Cr (mg/kg)	0.1 ± 0.2c	0.1 ± 0.1c	6.3 ± 0.2a	5.4 ± 0.2b
Pb (mg/kg)	0 ± 0b	0 ± 0b	0.7 ± 0.1a	0.7 ± 0.1a

^a^C:N, carbon-to-nitrogen ratio; NH_4_-N, ammonium-N; NO_3_-N, nitrate-N.

^b^Means followed by the same letter within a row do not differ (*P* < 0.05).

Total C and N contents in insect frass were higher than PL (Table 1; Supplementary Table 2), leading us to partially accept our first hypothesis. Particularly, IF-C had 93% and 60% higher total C and N than PL-AR, and an intermediate C:N ratio. Ammonium contents were higher in PL, while NO_3_-N contents were higher in insect frass. As such, both insect frass and PL were primarily composed of organic-N forms (97% and 73%, respectively). Phosphorus content in PL-AR was higher than insect frass, but P content in PL-NC was lower than IF-L and similar to IF-C. IF-L had the highest SRP content, corresponding to 58% of available P, whereas SRP comprised only 4%–8% of available P content in PL. Mg contents were higher in frass than in PL-AR. Contents of K, Ca, and S were also higher in PL than in frass, with the highest values observed for PL-AR (Table 1; Supplementary Table 2).

**Fig. 2.**
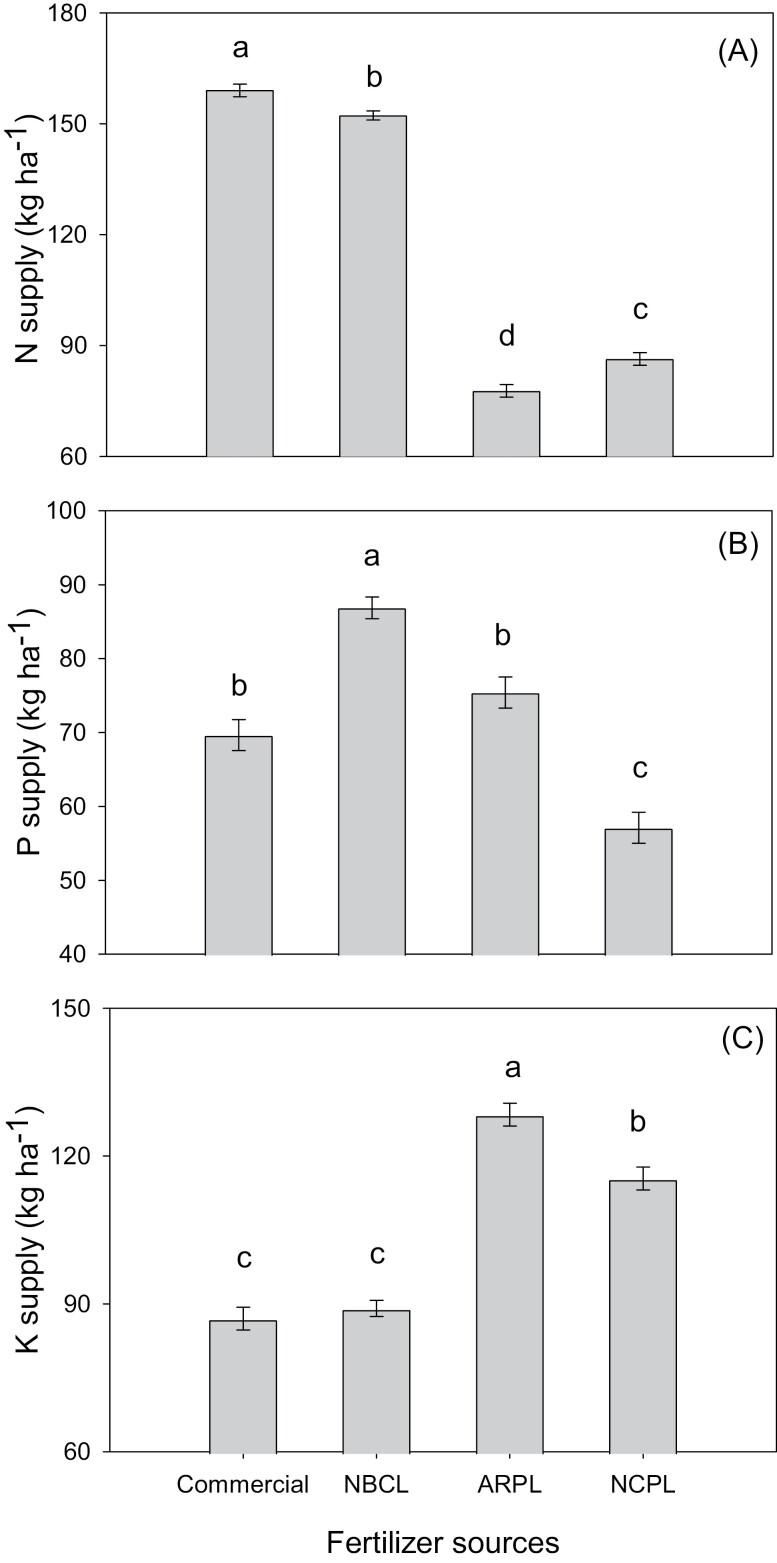
A) Nitrogen, B) P, and C) K supply (kg ha^-1^) of insect frass samples obtained from an insect-rearing company (IF-C) and the USDA ARS National Biological Control Laboratory (IF-L) and poultry litter samples collected in Arkansas (PL-AR) and North Carolina (PL-NC) at 5 Mg ha^-1^ application rate. Means (*n* = 3) followed by the same letter within the same nutrient (N, P, or K) do not differ (*P* < 0.05).

Nutrient supply was also affected by fertilizer source and production conditions (Supplementary Table 3). Considering a 5 Mg ha^-1^ application rate (Houben et al. 2021) and a moisture content of 10% for insect frass (Beesigamukama et al. 2022) and 30% for PL (Katuwal et al. 2023), IF-C can supply 159 kg ha^-1^ N, 5% higher than IF-L, 103% higher than PL-AR, and 85% higher than PL-NC (Fig. 2). In turn, the potential K supply of PL was, on average, 39% higher (122 kg ha^-1^ K) than in insect frass (88 kg ha^-1^ K). As for P, IF-L had a 25% greater P supply than IF-C (70 kg ha^-1^ P), 16% higher than PL-AR (75 kg ha^-1^ P), and 53% higher supply than PL-NC (57 kg ha^-1^).

PL had much higher contents of micronutrients, reaching 35, 3, 2, and 5 times greater mean Cu, Fe, Mn, and Zn contents than in insect frass, respectively (Table 1; Supplementary Table 2). Additionally, PL had, on average, 37 times more Na and 45 times more Al than insect frass. As for potentially toxic elements, insect frass had much lower Cd (0.2 mg/kg) and Cr (0.1 mg/kg) contents, and no As or Pb, leading us to partially accept our second hypothesis. As such, for a 5 Mg ha^-1^ application rate, micronutrient supply and heavy metal loads of PL (2 kg Cu, 0.8 kg Fe, 1.8 kg Mn, 2.3 kg Zn, 20 g Cr, 3.5 g As, and 2.5 g Pb per hectare) are much higher than that of insect frass (78 g Cu, 356 g Fe, 0.9 kg Mn, 0.5 kg Zn, and 0.5 g Cr per hectare).

### Indicator Microorganisms and Pathogens

Indicator microorganisms and major foodborne pathogens were less present in PL than in insect frass, although counts were generally low for both fertilizer sources and near the detection limit (Table 2; Supplementary Table 4). Aerobic bacterial counts in IF-C were 61% and 44% higher than IF-L and PL-AR, respectively. Similarly, *Enterobacteriaceae* counts in IF-C were 62% higher than IF-L, while undetected in PL-AR. Total coliforms in IF-C and IF-L were 192 and 78% higher than PL-AR, and *E. coli* counts were 62%–66% higher in frass than in PL-AR, respectively. Yeast and mold were 2.5 times higher in IF-C than in PL-AR and not detected in IF-L. Lactic acid bacteria counts in IF-C were 27% higher than IF-L and 79% higher than PL-AR. *Campylobacter* counts were similar in IF-C and PL-AR samples (2.7–2.85) and not detected in IF-L. Environmental *Listeria* and *Salmonella* were not detected in either frass or PL sources.

**Table 2.** Indicator microorganisms and major foodborne pathogens (mean ± standard error) in insect frass samples obtained from an insect-rearing company (IF-C), USDA-ARS National Biological Control Laboratory (IF-L), and poultry litter samples collected in Arkansas (PL-AR)

Pathogens	Fertilizer source			DL^a^
	IF-C	IF-L	PL-AR	
	Log_10_ CFU/g			
Aerobic bacterial counts	6.20 ± 0.06a^b^	3.85 ± 0.25b	4.30 ± 0.09b	1 Log
*Enterobacteriaceae*	5.17 ± 0.001a	3.19 ± 0.004b	ND^c^	1 Log
Total Coliforms	5.26 ± 0.016a	3.20 ± 0.008b	1.80 ± 0.3c	1 Log
*E. coli*	2.99 ± 0.11a	2.91 ± 0.025a	1.80 ± 0.1b	1 Log
*Campylobacter*	2.85 ± 0.05a	ND	2.70 ± 0.4a	2 Log
Environmental *Listeria*	ND	ND	ND	0.33 Log
*Salmonella*	ND	ND	ND	2 Log
Yeast and mold	3.90 ± 0.00a	ND	1.10 ± 0.00b	1 Log
Lactic acid bacteria	4.32 ± 0.02a	3.40 ± 0.2b	2.42 ± 0.01c	1 Log

^a^DL, detection limit.

^b^Means followed by the same letter within a row do not differ (*P* < 0.05).

^c^ND, not detectable.

## Discussion

The chemical characterization of this novel nutrient source is the first step toward the development of new value-added fertilizer products for this emerging mini livestock system. The positive correlation between P contents and Ca, Fe, and NH_4_-N and the negative correlation with C suggest that P is mostly associated with inorganic forms in these fertilizer sources. Still, this trend seems more prominent for insect frass, owing to increased SRP values (50%–58% of available P). We speculate that increased SRP levels in insect frass may be related to the content of SRP in wheat bran (0.3%), coupled with a lower P absorption efficiency by the insects. Soluble reactive P is highly bioavailable and can favor P uptake and crop nutrition, which is a positive agronomic attribute. However, SRP in organic fertilizer sources is directly linked to surface P runoff (Pote et al. 1996, DeLaune et al. 2004), leading to consequent pollution of water resources. Thus, further understanding of P chemical speciation is needed to assess the impacts of insect frass applications on soil and environmental health.

An increasing number of studies on the chemical characterization of insect frass have emerged in the past 5 yr, several of which focused on testing insect species and rearing substrates to assess the potential of frass as an alternative nutrient source. Houben et al. (2020) reported a total N content of 5% in frass of *T. molitor* larvae fed exclusively with wheat bran, higher than the observed in the present study, but similar C (39%), P (2%), and K (1.7%) contents. Beesigamukama et al. (2022) found that frass of *T. molitor* had much higher C/N ratio (20), higher total C (50%) and Ca contents (0.4%), lower N (2.5%) and P (1.4%) contents, but similar K (2%), Mg (0.6%), and S (0.3%) levels. In the latter study, larvae were fed with wheat bran and chayote (*Sechium edule*), which is a good source of carbohydrates but has lower protein and macronutrient contents compared with potatoes (USDA 2019). Except for Fe, the contents of micronutrients in the present study were similar to those obtained by Houben et al. (2020) and Beesigamukama et al. (2022). Differences in elemental composition were likely due to inherent diet nutritive values used in rearing conditions.

Both insect frass fertilizers in the present study have higher N and a similar P supply than that reported by Beesigamukama et al. (2022) (124 and 70 kg/ha, respectively) for *T. molitor*, as well as a similar supply of micronutrients. Application rates of *T. molitor* frass still need to be developed and tested in field conditions for commercial crops, so the agronomic efficiency of insect frass can be compared with other manures and synthetic mineral fertilizers. As most N in the studied insect frass was found in organic forms, N mineralization rates and availability would be limited, and a 5 Mg/ha application may be insufficient to supply adequate N crops (Houben et al. 2021). The authors argued that 10 Mg/ha of *T. molitor* frass was needed to increase N uptake and biomass of Italian ryegrass (*Lolium multiflorum* L.) compared with mineral fertilizer. Doubling the insect frass application rate can not only improve N and micronutrient supply but also increase the risk of P runoff, thus highlighting the need for proper insect frass application rates.

Considering the same application rate, insect frass would supply more N and P than PL, although PL is undoubtedly a better source of micronutrients. With the projected annual 28% increase in the insect farming sector by 2030 (Meticulous Research 2023), insect frass production is expected to expand, leading to higher product availability and reduction in prices. For instance, a commercial facility can produce 635 kg of insect frass weekly, taking up approximately 1,400 kg of organic waste, which agrees with the 50% bioconversion factor demonstrated by Wang et al. (2017). Repurposing organic waste is a central focus of circular economies along with reducing CH_4_ and CO_2_ emissions and contamination of soil and groundwater. As such, insect frass is an environmentally friendly, sustainable fertilizer source, which can be used as a partial replacement of mineral fertilizers or as a complement for PL applications, thus enhancing organic matter and macronutrient contents in a micronutrient-enriched material and reducing the concentration of PTEs, as discussed in the next section.

To the best of our knowledge, this study was the first to demonstrate the virtual absence of heavy metals in insect frass, which is likely due to the composition of the rearing substrate. In contrast, the higher contents of Al, Na, and heavy metals in PL are reflective of amendments and growth-promoting supplements added to the poultry feed (Ashworth et al. 2020, Katuwal et al. 2023). Considering the low or null levels of heavy metals, insect frass seems a safer fertilizer source compared with PL. In addition, insect frass can be used as a soil amendment to complex heavy metals, such as Cd and Ni (Watson et al. 2021), thereby reducing their bioavailability and potential environmental contamination.

Greater counts of indicator microorganisms and foodborne pathogens in the commercial frass compared with IF-L and PL sources reflect differences in insect-rearing conditions, namely feed sources. Specifically, NBCL used food-grade bran and dry potatoes as substrate, while the commercial facility included dry and wet organic waste. Yet, the counts were very low, below the values recommended by the National Organic Program standards for compost (1,000 most probable number [MPN] fecal coliform per gram, or more than 3 MPN Salmonella per 4 g; National Organic Standards Board 2006), and the presence of indicator microorganisms and *Campylobacter*, a major foodborne pathogen, should not pose risk to environmental and human health. Additional amendments or treatments can be applied to further lower the microbial load. Recently, Van Looveren et al. (2022) tested the impact of heat treatment (70 °C for 60 min) on the microbiological quality and safety of *H. illucens* (black soldier fly) and demonstrated that the treatment reduced Enterobacteriaceae, *Salmonella* spp., and *Clostridium perfringens* to values below detection limits (1–2 Log_10_ CFU/g). Regulations for indicator microorganisms and pathogens in insect frass are still developing, as well as definitions and allowances for the use of insect frass in agriculture (USDA NOP 2016, OJEU 2021); thus, maintenance of low microbial counts via heat treatment may be a good strategy for allowing the safe use of insect frass in agriculture. However, physicochemical property changes should be evaluated based on frass heat treatment.

Here, we analyzed the elemental composition and the presence of pathogens in insect frass compared with PL. Greater total N and soluble P and the virtual absence of heavy metals make insect frass a valuable, value-added fertilizer source. Organic N was the prevalent form in insect frass (97% of total N) and would likely affect N mineralization and bioavailability during year–1 of application; however, field studies are needed to confirm this result. Similarly, the greater proportion of soluble P (50%–58% of available P) would not only benefit P uptake and crop nutrition but also increase the risk of P runoff. As such, further investigation on P chemical forms is needed to evaluate the actual nutrient supply and potential environmental risks associated with applications of insect frass to soils. In addition, as the insect farming industry expands, microbial safety standards and guidelines need to be developed to ensure the production of a cleaner insect frass.

Compared with PL, insect frass had a superior supply of N and P, but a much lower supply of micronutrients. As such, future research testing different mealworm diets as well as combinations of fertilizer sources (e.g., insect frass + PL or insect frass + mineral fertilizer) would benefit the development of a more enriched, cheaper, and cleaner fertilizer, thus reducing production costs while minimizing the impacts associated with excessive use of synthetic fertilizers and waste disposal in the environment. Indeed, mixtures of insect frass and synthetic N–P–K fertilizers have been demonstrated to enhance soil fertility, plant biomass, and nutrient uptake to similar levels of synthetic fertilizers (Houben et al. 2020, Tanga et al. 2022). Moreover, field experiments including various commercial crops and application rates would shed light on how insect frass applications affect soil health, plant productivity, and crop nutrition. As a disruptive, yet still emerging technology, the use of insect frass is promising and aligns with global initiatives to promote more efficient food systems and sustainable intensification in agriculture.

## Supplementary Material

Supplementary material is available at *Journal of Economic Entomology* online.

toad234_suppl_Supplementary_Tables_1-4
