# Supplementary material for: Insect frass composition and potential use as an organic fertilizer in circular economies
Source: J Econ Entomol. 2024 Jan 3;117(4):1261–8. doi: 10.1093/jee/toad234 (PMC11318615; doi:10.1093/jee/toad234)
Supplement: toad234_suppl_Supplementary_Tables_1-4 [file toad234_suppl_supplementary_tables_1-4.docx]

**Supplementary Table 1.** Pearson’s correlation coefficients (*r*) between chemical properties of fertilizer sources [insect frass samples obtained from an insect rearing company (IF-C) and the USDA ARS National Biological Control Laboratory (IF-L) and poultry litter samples collected in Arkansas (PL-AR) and North Carolina (PL-NC)].

|  | **EC** | **N** | **C** | **C:N** | **NH_4_-N** | **NO_3_-N** | **SRP** | **Al** | **As** | **Ca** | **Cd** | **Cr** | **Cu** | **Fe** | **K** | **Mg** | **Mn** | **Na** | **P** | **Pb** | **S** | **Zn** |
| --- | --- | --- | --- | --- | --- | --- | --- | --- | --- | --- | --- | --- | --- | --- | --- | --- | --- | --- | --- | --- | --- | --- |
| pH | -0.26 | -0.97*** | -0.79*** | 0.08 | 0.66** | -0.79*** | -0.92*** | 0.88*** | 0.87*** | 0.95*** | 0.74*** | 0.98*** | 0.97*** | 0.82*** | 0.98*** | -0.58** | 0.91*** | 0.98*** | 0.40 | 0.96*** | 0.84*** | 0.97*** |
| EC |  | 0.17 | 0.09 | -0.17 | 0.21 | 0.28 | 0.01 | -0.33 | -0.31 | -0.04 | 0.06 | -0.14 | -0.24 | -0.13 | -0.12 | -0.27 | 0.10 | -0.18 | -0.24 | -0.15 | 0.11 | -0.18 |
| N |  |  | 0.85*** | -0.03 | -0.75*** | 0.70** | 0.91*** | -0.82*** | -0.79*** | -0.98*** | -0.77*** | -0.99*** | -0.94*** | -0.87*** | -0.98*** | 0.63** | -0.94*** | -0.96*** | -0.48 | -0.93*** | -0.90*** | -0.95*** |
| C |  |  |  | 0.50* | -0.94*** | 0.38 | 0.66** | -0.47 | -0.47 | -0.90*** | -0.93*** | -0.83*** | -0.69** | -0.91*** | -0.86*** | 0.78*** | -0.88*** | -0.73** | -0.77*** | -0.76*** | -0.95*** | -0.71** |
| C:N |  |  |  |  | -0.57* | -0.40 | -0.22 | 0.45 | 0.39 | -0.12 | -0.51* | 0.05 | 0.22 | -0.30 | -0.03 | 0.46 | -0.14 | 0.18 | -0.64** | 0.08 | -0.34 | 0.20 |
| NH_4_-N |  |  |  |  |  | -0.18 | -0.57* | 0.29 | 0.29 | 0.84*** | 0.90*** | 0.73*** | 0.55* | 0.85*** | 0.77*** | -0.79*** | 0.86*** | 0.60** | 0.75*** | 0.63** | 0.96*** | 0.58* |
| NO_3_-N |  |  |  |  |  |  | 0.87*** | -0.94*** | -0.93*** | -0.64** | -0.45 | -0.76*** | -0.88*** | -0.46 | -0.72*** | 0.39 | -0.60** | -0.85*** | 0.17 | -0.83*** | -0.44 | -0.87*** |
| SRP |  |  |  |  |  |  |  | -0.92*** | -0.90*** | -0.90*** | -0.66** | -0.95*** | -0.97*** | -0.72*** | -0.93*** | 0.63** | -0.90*** | -0.97*** | -0.11 | -0.95*** | -0.78*** | -0.98*** |
| Al |  |  |  |  |  |  |  |  | 0.98*** | 0.75*** | 0.44 | 0.86*** | 0.96*** | 0.57* | 0.83*** | -0.37 | 0.71*** | 0.94*** | -0.02 | 0.90*** | 0.55* | 0.95*** |
| As |  |  |  |  |  |  |  |  |  | 0.74*** | 0.44 | 0.85*** | 0.95*** | 0.53* | 0.83*** | -0.39 | 0.70** | 0.93*** | -0.01 | 0.92*** | 0.55* | 0.94 |
| Ca |  |  |  |  |  |  |  |  |  |  | 0.84*** | 0.98*** | 0.91*** | 0.89*** | 0.99*** | -0.74*** | 0.99*** | 0.93*** | 0.52* | 0.93*** | 0.96*** | 0.92*** |
| Cd |  |  |  |  |  |  |  |  |  |  |  | 0.77*** | 0.64** | 0.90*** | 0.80*** | -0.76*** | 0.85*** | 0.69** | 0.62** | 0.73*** | 0.90*** | 0.66** |
| Cr |  |  |  |  |  |  |  |  |  |  |  |  | 0.97*** | 0.86*** | 0.99*** | -0.65** | 0.96*** | 0.98*** | 0.40 | 0.97*** | 0.89*** | 0.98*** |
| Cu |  |  |  |  |  |  |  |  |  |  |  |  |  | 0.74*** | 0.95*** | -0.57* | 0.87*** | 1.00*** | 0.22 | 0.97*** | 0.76*** | 1.00*** |
| Fe |  |  |  |  |  |  |  |  |  |  |  |  |  |  | 0.86*** | -0.60** | 0.89*** | 0.77*** | 0.66** | 0.76*** | 0.90*** | 0.75*** |
|  | **EC** | **N** | **C** | **C:N** | **NH_4_-N** | **NO_3_-N** | **SRP** | **Al** | **As** | **Ca** | **Cd** | **Cr** | **Cu** | **Fe** | **K** | **Mg** | **Mn** | **Na** | **P** | **Pb** | **S** | **Zn** |
| K |  |  |  |  |  |  |  |  |  |  |  |  |  |  |  | -0.68** | 0.97*** | 0.97*** | 0.46 | 0.97*** | 0.92*** | 0.96*** |
| Mg |  |  |  |  |  |  |  |  |  |  |  |  |  |  |  |  | -0.75*** | -0.60** | -0.33 | -0.65** | -0.79*** | -0.59** |
| Mn |  |  |  |  |  |  |  |  |  |  |  |  |  |  |  |  |  | 0.90*** | 0.47 | 0.90*** | 0.97*** | 0.89*** |
| Na |  |  |  |  |  |  |  |  |  |  |  |  |  |  |  |  |  |  | 0.26 | 0.98*** | 0.80*** | 1.00*** |
| P |  |  |  |  |  |  |  |  |  |  |  |  |  |  |  |  |  |  |  | 0.28 | 0.64** | 0.22 |
| Pb |  |  |  |  |  |  |  |  |  |  |  |  |  |  |  |  |  |  |  |  | 0.82*** | 0.98*** |
| S |  |  |  |  |  |  |  |  |  |  |  |  |  |  |  |  |  |  |  |  |  | 0.79*** |
| EC, electrical conductivity; C:N, carbon-to-nitrogen ratio; NH_4_-N, ammonium-N; NO_3_-N, nitrate-N; SRP, soluble reactive P;  ***, *p* < 0.001; **, *p* < 0.01; *, *p* < 0.05. | | | | | | | | | | | | | | | | | | | | | | |

**Supplementary Table 2.** Analysis of variance summary of chemical composition (response variable) of fertilizer sources [insect frass samples obtained from an insect rearing company (IF-C) and the USDA ARS National Biological Control Laboratory (IF-L) and poultry litter samples collected in Arkansas (PL-AR) and North Carolina (PL-NC)]. Fertilizer source (IF-C, IF-L, AR-PL, and NC-PL) was considered the main effect (explanatory variable) and replicate samples (*n* = 3) as random in a completely randomized design

| **Chemical properties†** | **Num DF** | **F** | **P** |
| --- | --- | --- | --- |
| pH | 3 | 53.78 | <0.0001 |
| EC | 3 | 23.03 | <0.0001 |
| C | 3 | 192.23 | <0.0001 |
| C:N | 3 | 11.94 | 0.0009 |
| N | 3 | 210.36 | <0.0001 |
| NH_4_-N | 3 | 2886.49 | <0.0001 |
| NO_3_-N | 3 | 31.82 | <0.0001 |
| Organic N | 3 | 250.60 | <0.0001 |
| P | 3 | 33.71 | <0.0001 |
| SRP | 3 | 1040.67 | <0.0001 |
| K | 3 | 287.98 | <0.0001 |
| Ca | 3 | 1507.06 | <0.0001 |
| Mg | 3 | 5.08 | 0.0190 |
| S | 3 | 690.07 | <0.0001 |
| Al | 3 | 315.56 | <0.0001 |
| Na | 3 | 1230.43 | <0.0001 |
|  | **Num DF** | **F** | **P** |
| Cu | 3 | 735.36 | <0.0001 |
| Fe | 3 | 21.38 | <0.0001 |
| Mn | 3 | 701.48 | <0.0001 |
| Zn | 3 | 878.39 | <0.0001 |
| As | 3 | 80.75 | <0.0001 |
| Cd | 3 | 21.80 | <0.0001 |
| Cr | 3 | 557.73 | <0.0001 |
| Pb | 3 | 71.65 | <0.0001 |
| † EC, electrical conductivity; C:N, carbon-to-nitrogen ratio; NH_4_-N, ammonium-N; NO_3_-N, nitrate-N; SRP, soluble reactive P; | | | |

**Supplementary Table 3.** Analysis of variance summary of nutrient supply (response variable) of fertilizer sources [insect frass samples obtained from an insect rearing company (IF-C) and the USDA ARS National Biological Control Laboratory (IF-L) and poultry litter samples collected in Arkansas (PL-AR) and North Carolina (PL-NC)]. Fertilizer source (IF-C, IF-L, AR-PL, and NC-PL) was considered the main effect (explanatory variable) and replicate samples (*n* = 3) as random in a completely randomized design.

| **Nutrient supply** | **Num DF** | **F** | **P** |
| --- | --- | --- | --- |
| N | 3 | 724.76 | <.0001 |
| P | 3 | 47.76 | <.0001 |
| K | 3 | 88.73 | <.0001 |

**Supplementary Table 4.** Analysis of variance summary of bacterial counts (response variable) of fertilizer sources [insect frass samples obtained from an insect rearing company (IF-C) and the USDA ARS National Biological Control Laboratory (IF-L), and poultry collected in Arkansas (PL-AR)]. Fertilizer source (IF-L, IF-C, and AR-PL) was considered the main effect (explanatory variable) and replicate samples (*n* = 3) as random in a completely randomized design.

| **Pathogens** | **Num DF** | **F** | **P** |
| --- | --- | --- | --- |
| Aerobic Bacterial Counts | 2 | 62.9 | 0.0036 |
| Enterobacteriaceae | 2 | 1095536 | <0.0001 |
| Total Coliforms | 2 | 100.5 | 0.0018 |
| Escherichia coli | 2 | 57.93 | 0.004 |
| Campylobacter | 2 | 4.815 | 0.1158 |
| Environmental Listeria | 2 | - | - |
| Salmonella | 2 | - | - |
| Yeast and Mold | 2 | 112533 | <0.0001 |
| Lactic Acid Bacteria | 2 | 66.87 | 0.0032 |
